# Strategies to produce T-DNA free CRISPRed fruit trees via *Agrobacterium tumefaciens* stable gene transfer

**DOI:** 10.1038/s41598-020-77110-1

**Published:** 2020-11-19

**Authors:** Lorenza Dalla Costa, Stefano Piazza, Valerio Pompili, Umberto Salvagnin, Alessandro Cestaro, Loredana Moffa, Lorenzo Vittani, Claudio Moser, Mickael Malnoy

**Affiliations:** Research and Innovation Centre, Fondazione Edmund Mach, via E. Mach 1, 38098 San Michele all’Adige, Italy

**Keywords:** Biotechnology, Molecular biology, Plant sciences

## Abstract

Genome editing via CRISPR/Cas9 is a powerful technology, which has been widely applied to improve traits in cereals, vegetables and even fruit trees. For the delivery of CRISPR/Cas9 components into dicotyledonous plants, *Agrobacterium tumefaciens* mediated gene transfer is still the prevalent method, although editing is often accompanied by the integration of the bacterial T-DNA into the host genome. We assessed two approaches in order to achieve T-DNA excision from the plant genome, minimizing the extent of foreign DNA left behind. The first is based on the Flp/FRT system and the second on Cas9 and synthetic cleavage target sites (CTS) close to T-DNA borders, which are recognized by the sgRNA. Several grapevine and apple lines, transformed with a panel of CRISPR/SpCas9 binary vectors, were regenerated and characterized for T-DNA copy number and for the rate of targeted editing. As detected by an optimized NGS-based sequencing method, trimming at T-DNA borders occurred in 100% of the lines, impairing in most cases the excision. Another observation was the leakage activity of Cas9 which produced pierced and therefore non-functional CTS. Deletions of genomic DNA and presence of filler DNA were also noticed at the junctions between T-DNA and genomic DNA. This study proved that many factors must be considered for designing efficient binary vectors capable of minimizing the presence of exogenous DNA in CRISPRed fruit trees.

## Introduction

Over the last few years, the development of genome editing techniques boosted green biotechnology research committed to crop improvement. Editing technology, based predominantly on CRISPR systems, has been applied to various crop species offering tremendous opportunities for trait improvement in vegetatively propagated crops, which can thus conserve the overall genetic make-up^1,2^. However, this huge biotechnological potential is sometimes hampered by legislative boundaries which regulate agricultural production at a national level.

USA, Argentina, Brazil and Chile have established that if no foreign genes or genetic material are present in the edited plant variety, the additional regulatory oversight and risk assessment for GMO products will not apply^3^. Other countries, such as Australia and New Zealand, are still examining the regulation of new breeding technologies^4^. Conversely, Europe, on 25 July 2018, strongly reaffirmed the precautionary principle (PP) on this matter. The European Court of Justice ruled out that organisms obtained by new techniques of directed mutagenesis are genetically modified organisms (GMOs) and as such must comply with the demanding provisions of the Directive 2001/18/EC concerning the deliberate release into the environment of GMOs. However, one of the pillars of the PP, defined by the European Commission (EC) in 2000, is the revision of the measures in light of scientific developments^5^. As argued by the Group of Scientific Advise Mechanism (SAM) of the EC^6^, it is evident that the huge scientific and technological developments of the past twenty years (the law dates from 2001) have made the GMO Directive no longer fit for purpose. One of the biggest concerns regards the impossibility of distinguishing between spontaneously occurring mutations and different types of human interventions (random or directed mutagenesis) thus undermining the foundation of the principles of traceability and labelling underpinning the European legislation on GMO^7^. It is therefore conceivable that in Europe the regulatory framework for GMO will put more emphasis on the features of the end product rather than on the technique of production, aligning with the prevalent approach worldwide, as advocated by SAM and by the European scientific community.

Unlike the legislative process, the research in the field of genome editing is proceeding at a fast pace and advancements to avoid the presence of foreign DNA have been reported. Besides methods based on *Agrobacterium tumefaciens* (*A.t.*) where stable integration of exogenous DNA is followed by backcrossing (only feasible for sexually propagated annual plants), transient expression of plasmids carrying CRISPR components^8^ or methods relying on the delivery of CRISPR/Cas9 protein-RNA complex (RNP) to protoplasts^2^ are being developed.

Nonetheless, *Agrobacterium*-mediated stable gene transfer is still the prevalent method of genetic engineering in dicotyledonous plants, despite the drawback of T-DNA integration in the host genome. T-DNA or transferred DNA is the DNA segment carrying all the exogenous elements that will be transferred in the plant genome. The processing of T-DNA and its subsequent export from the bacterium to the plant cell results in large part from the activity of virulence (*vir*) genes carried by the T-helper plasmid^9^. T-DNA has two sequences at its borders: left border (LB) and right border (RB), 24 bp (nopaline) or 25 bp (octopin) long, highly homologous and arranged in a directly repeated orientation. These are the target of the VirD1/VirD2 endonuclease complex that processes T-DNA by nicking the “lower strand” between nucleotides 3 and 4 of the border sequences, shortening LB by 3 bp and RB by 22 bp^10^. The resulting T-strand is then transported in the plant cell and integrated randomly into the plant genome, in prevalence in those temporary DNA holes named double strand breaks (DSB).

In this study we applied *Agrobacterium*-mediated transformation on two economically important fruit trees, grapevine and apple, and evaluated different constructs for the complete elimination of exogenous DNA via site-specific removal mechanisms. We designed vectors carrying the CRISPR/Cas9 system to knock-out the genes *VviMLO7* and *MdDIPM1* and *MdDIPM4* which have a role in the susceptibility to powdery mildew in grapevine^11^ and fire blight in apple^12^, respectively. Two mechanisms were tested for the removal of T-DNA. One is based on the site-specific recombinase Flippase *(Flp)* which recognizes the 34-bp long FRT sequences. The Flp/FRT system has been extensively used to remove undesired transgenes from transgenic apple^13,14^ and grapevine^15^. Recently in Pompili et al.^12^ it has been successfully applied to remove T-DNA cassette in apple. This latter work can be considered as a starting point for our study which basically consists in the optimization of vectors for T-DNA free genome editing. The second removal mechanism relies on the Cas9 enzyme cleavage mechanism. We decided to exploit the cleavage activity of Cas9 not only to edit the endogenous target site, but also to remove T-DNA by placing two additional synthetic target sites (named cleavage target sites, CTS) next to the LB and RB sites. In order to assess the presence and length of exogenous DNA in the genome of the edited plants, a rapid NGS method has been set up. This method has been efficient in elucidating the integration of T-DNA at base pair resolution, as well as the integrity of the border neighboring regions of the host genome.

## Results

### Design of vectors with different mechanisms for T-DNA site-specific removal

**Figure 1:**
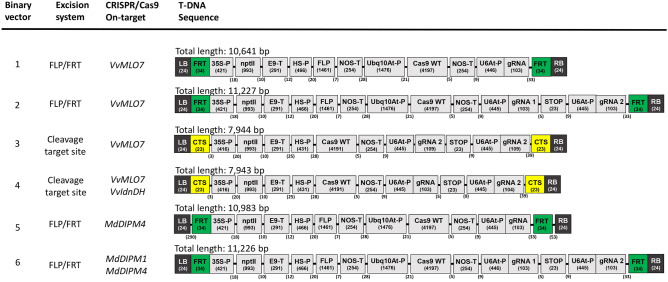
Summary of binary vectors carrying self-excisable CRISPR T-DNAs used in the present study. Vectors from 1 to 4 were used for grapevine transformation while vectors 5 and 6 for apple transformation. T-DNAs contained the CRISPR/Cas9 system driven by a constitutive (*Arabidopsis thaliana Ubiquitin-10* Promoter, Ubq10At-P) or an inducible (Heat Shock-Promoter, HS-P) promoter. For grapevine, the editing targets were the powdery mildew susceptibility gene *VvMLO7* and the *L-idonate dehydrogenase* gene *VvIdnDH*. For apple, the editing targets were the fire blight susceptibility genes *MdDIPM1* and *MdDIPM4*. T-DNAs were specifically designed to be self-excisable by using two different excision systems: (i) the FLP (*Flippase*)/FRT (Flippase Recognition Target site) recombination system and (ii) the CTS (Cleavage Target Site) recognized by the CRISPR/Cas9 system. Left and Right Borders (LB and RB); *Cauliflower Mosaic Virus 35S* Promoter (35S-P); *Neomycin phosphotransferase II* (*nptII*); E9 Terminator (E9-T); *NopalinE Synthase* Terminator (NOS-T); *Crispr associated protein 9 wild-type* (Cas9 WT); *Arabidopsis thaliana U6* Promoter (U6At-P); guide RNA for the CRISPR/Cas9 system (gRNA); short hairpin to detach RNA-polymerase from DNA strand (STOP).

**Figure 2:**
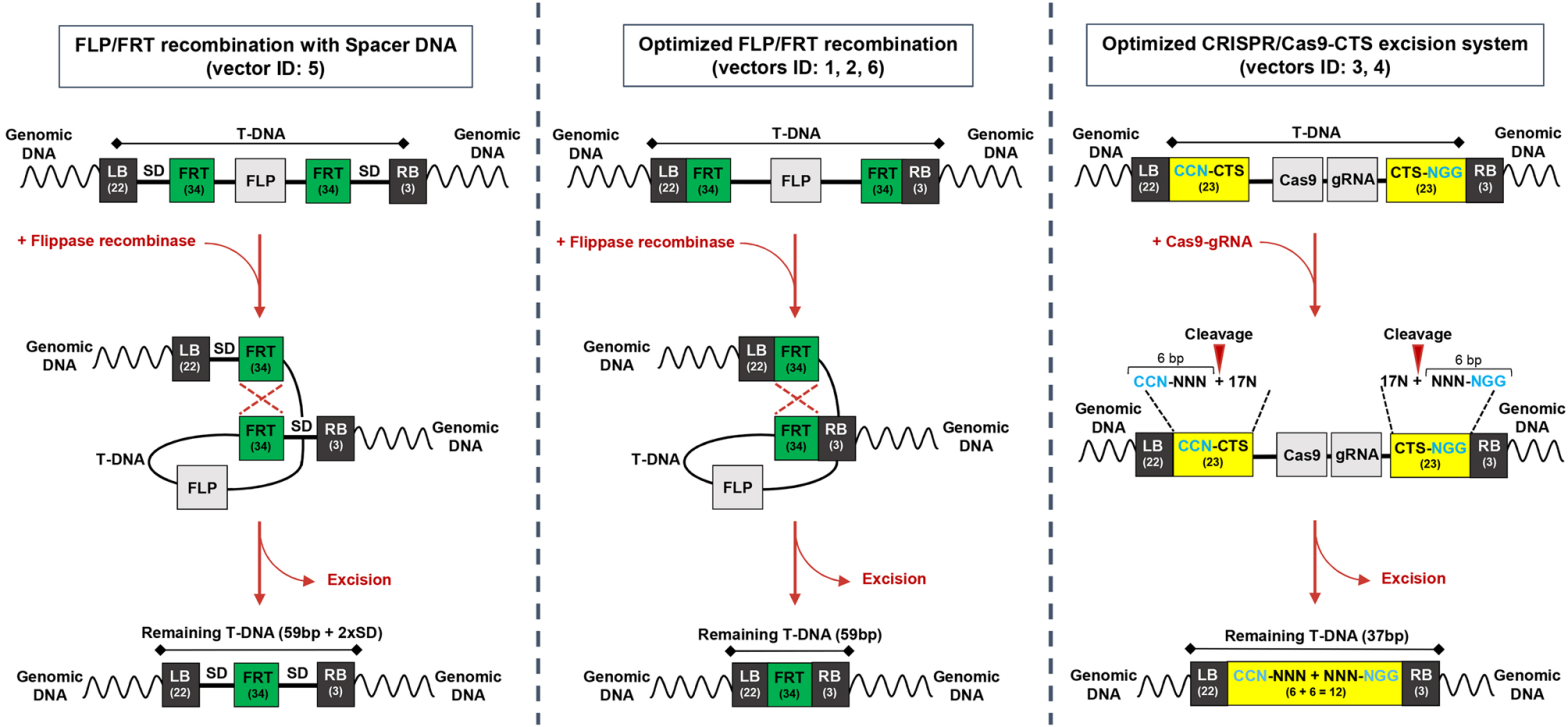
Schematic representation of T-DNA excision mechanisms exploited by the binary vectors used in this study. See Fig. 1 for the legend. Spacer DNA (SD).

In total, six binary vectors were designed and used in this study in order to assess the best strategy to achieve gene editing of susceptibility genes and removal of the T-DNA cassette (Fig. 1). Vectors 1–4 were used in grapevine to edit *VvMLO7* gene and vectors 5 and 6 in apple to edit members of MdDIPM gene family. In vectors 1, 2, 5 and 6 the mechanism for the removal of T-DNA is based on the site-specific recombinase *Flp* and the related FRT sequences. While in vector 5 there is a spacer DNA between LB and FRT (290 bp) and between FRT and RB (53 bp), in vectors 1, 2 and 6, these elements are seamless at the borders (Fig. 1). Furthermore, vectors 3 and 4 exploit the cleavage activity of Cas9, which recognizes two synthetic target sites (CTS), consisting of the same sequence but inverted, located next to LB and RB without spacer DNA in between (Fig. 1 and Fig. 2). In vector 3, CTS is the 20-bp sequence of *VvMLO7* gene recognized by the guide RNA-*Mlo7*, while in vector 4 CTS is the 20-bp sequence of grapevine L-idonate dehydrogenase gene (*VvIdnDH*) recognized by the guide RNA-*VvIdnDH*. This target site has been chosen as the one reported in the first paper describing a successful application of CRISPR/Cas9 in grapevine^16^. In addition to the 20 nt-sequence identical to the endogenous target, CTSs also include the PAM site in order to allow the Cas9 cleavage (Fig. 2). Overall, both the excision systems have been placed under the control of an inducible promoter, the soybean heat shock promoter which was previously used for the removal of selection marker genes for cisgenesis in grapevine and apple^13,15^. The availability of an inducible system for T-DNA cassette excision is crucial since the induction step should be strictly controlled in time.

### Gene transfer, induction of T-DNA removal and evaluation of editing

Gala and Golden Delicious varieties were used for apple transformation, while Chardonnay, Thompson Seedless, Sugraone and the model genotype Microvine, for grapevine. Several *Agrobacterium* gene transfer experiments were carried out and T-DNA molecular features of the regenerated lines, 9 for apple and 14 for grapevine, are reported in Table 1. The number of T-DNA integrations in the plant genome, evaluated by quantifying *nptII* or *Cas9* copy number (CN), were all very close to a single copy except for lines V4-34 and GT92.2 that were closer to 2. The first regenerated lines were heat-shock induced by adopting different approaches (strategies A, B, C, Table 1), whereas those subsequently obtained were at first analyzed with the NGS method to check the integrity of T-DNA extremities. As reported in Table 1, a few induced biological replicates of apple showed a CN significantly lower compared to that of the not-induced mother plant (lines transformed with vector 5) while in the case of grapevine lines, no significant lower CN was detected after heat-shock treatment.

**Table 1:** Assessing T-DNA excision and targeted editing detection in grapevine and apple transgenic lines.

Vector	Genotype	plant ID	Copy Number CN n = nptII; c = Cas9	CN after induction A = induced plant 6 h. 42 °C 3x; B = induced bud 3 h. 42 °C 3x; C = induced bud 3 h. 42 °C 5x	Cas9 induction	Targeted editing percentage
***Vitis vinifera***						
Vector 1	Chardonnay	GT89.3	0.71 ± 0.22 (n)	n.i.	Constitutive	50%
	Thompson seedless	GT90.1	0.92 ± 0.06 (n)	A 0.98 ± 0.09 (n)	Constitutive	0%
Vector 2	Microvine	GT92.2	1.81 ± 0.09 (n)	n.i.	Constitutive	n.a.
		GT103.1	0.97 ± 0.17(n)	B_1_ 0.83; B_2_ 0.84; C 0.87	Constitutive	B_1_ 4.8% + 5.6%; B_2_ 5% + 5.3%; C 3% + 3%
		GT103.2	0.85 ± 0.04 (n)	C 0.86	Constitutive	C 16.6% + 14%
Vector 3	Sugraone	GT109.3	0.85 (c)	A 0.75 ± 0.13	Inducible	B 1.4%
Vector 4	Sugraone	GT110.4	1.2 ± 0.4 (c )	A_1_ 0.78; A_2_ 0.83; A_3_ 0.67; C_1_ 0.96; C_2_ 0.92	Inducible	A_1_ 0%; A_2_ 0%; A_3_ 0%; C_1_ 0%; C_2_ 0%
		GT110.5	0.8 ± 0.25 (c )	A_1_ 0.85; A_2_ 0.73; C_1_ 0.82; C_2_ 0.71	Inducible	A_1_ 12%; A_2_ 29%; C_1_ 6%; C_2_ 6.6%
		GT110.6	0.79 ± 0.13 (c )	A_1_ 0.93; A_2_ 0.79; C_1_ 0.88; C_2_ 0.92	Inducible	A_1_ 5%; A_2_ 3.3%; C_1_ 9%; C_2_ 4%
		GT110.8	0.82 (c )	A 0.85	Inducible	A 0%
		GT110.11	1.14 (c )	n.i.	Inducible	n.a.
		GT110.15	1.34 (c )	n.i.	Inducible	n.a.
		GT110.18	0.78 (c )	n.i.	Inducible	n.a.
		GT110.20	1.24 (c )	n.i.	Inducible	n.a.
***Malus x domestica***						
Vector 5	Gala	V1-4	1.1 (n)	n.i.	Constitutive	0%
		V1-10	0.9 ± 0.1 (n)	A_1_ 0.61; A_2_ 0.15; A_3_ 0.2; A_4_ 1.14; A_5_ 0.48; A_6_ 0.07; A_7_ 0.13; A_8_ 0.26; A_9_ 0.3; A_10_ 0.3	Constitutive	100%
		V1-14	0.6 (n)	n.i.	Constitutive	50%
	Golden Delicious	V2-3	1.0 ± 0.1(n)	A_1_ 0.13; A_2_ 0.25; A_3_ 0.62; A_4_ 0.74; A_5_ 0.32; A_6_ 0.67; A_7_ 0.1; A_8_ 0.28; A_9_ 0.11; A_10_ 0.63	Constitutive	100%
		V4-5	0.6 (n)	n.i.	Constitutive	0%
		V4-27	0.8 (n)	n.i.	Constitutive	100%
		V4-34	1.6 ± 0.3 (n)	A_1_ 1.35; A_2_ 1.48; A_3_ 1.98; A_4_ 1.77; A_5_ 1.4; A_6_ 1.7; A_7_ 1.63; A_8_ 1.64; A_9_ 1.76; A_10_ 0.0	Constitutive	100%
Vector 6	Gala	V6-2	1.10 ± 0.12 (n)	n.i.	Constitutive	100%
		V6-10	0.95 ± 0.13 (n)	n.i.	Constitutive	100%

Using the binary vectors illustrated in Fig. 1, fourteen grapevine and nine apple transgenic lines, from different genotypes, were produced and included in the present study. The presence of T-DNA in the genome of not-induced and heat-shock induced (via the strategies A, B, C) plants was evaluated by quantifying the *nptII* (n) or *Cas9* (c) copy number (CN) using a Taqman Real time-PCR method described in the Materials and Methods Section. CRISPR/Cas9 on-target editing was detected on the predicted target site by NGS as reported in the Materials and Methods Section. Not induced (n.i.); not applicable (n.a.). Data related to vector 5 are the one reported in Pompili et al.^12^.

The analysis of editing indicated both a complete and a partial editing depending on the lines. In particular, a complete (100%) or 50% editing was obtained only if the expression of Cas9 was constitutive (Table 1). When Cas9 was under the control of a heat-shock inducible promoter, a variable degree of editing was observed, ranging from 1.4% (line 109.3) to 29% (line 110.5) with a mean value around 10%, and no editing at all in two cases (lines 110.4 and 110.8). The highest level of editing (29%) was achieved in response to the induction of a plantlet at 42 °C for 6 h repeated for 3 times (line GT110.5 replicate A_2_). Regarding the type of mutations detected in apple, deletion is the most common, ranging from − 1 to − 7 bp. In grapevine, a single nucleotide insertion is the most common variation, most frequently Thymine followed by Adenine. In the case of the double target for the gene *VvMLO7* (lines GT103.1 and GT103.2), a deletion of the sequence between the two cleavage positions (33-bp long) was observed. Editing at the target site in *VvIdnDH* was not analyzed.

### Method to identify T-DNA integration points in the plant genome

**Figure 3:**
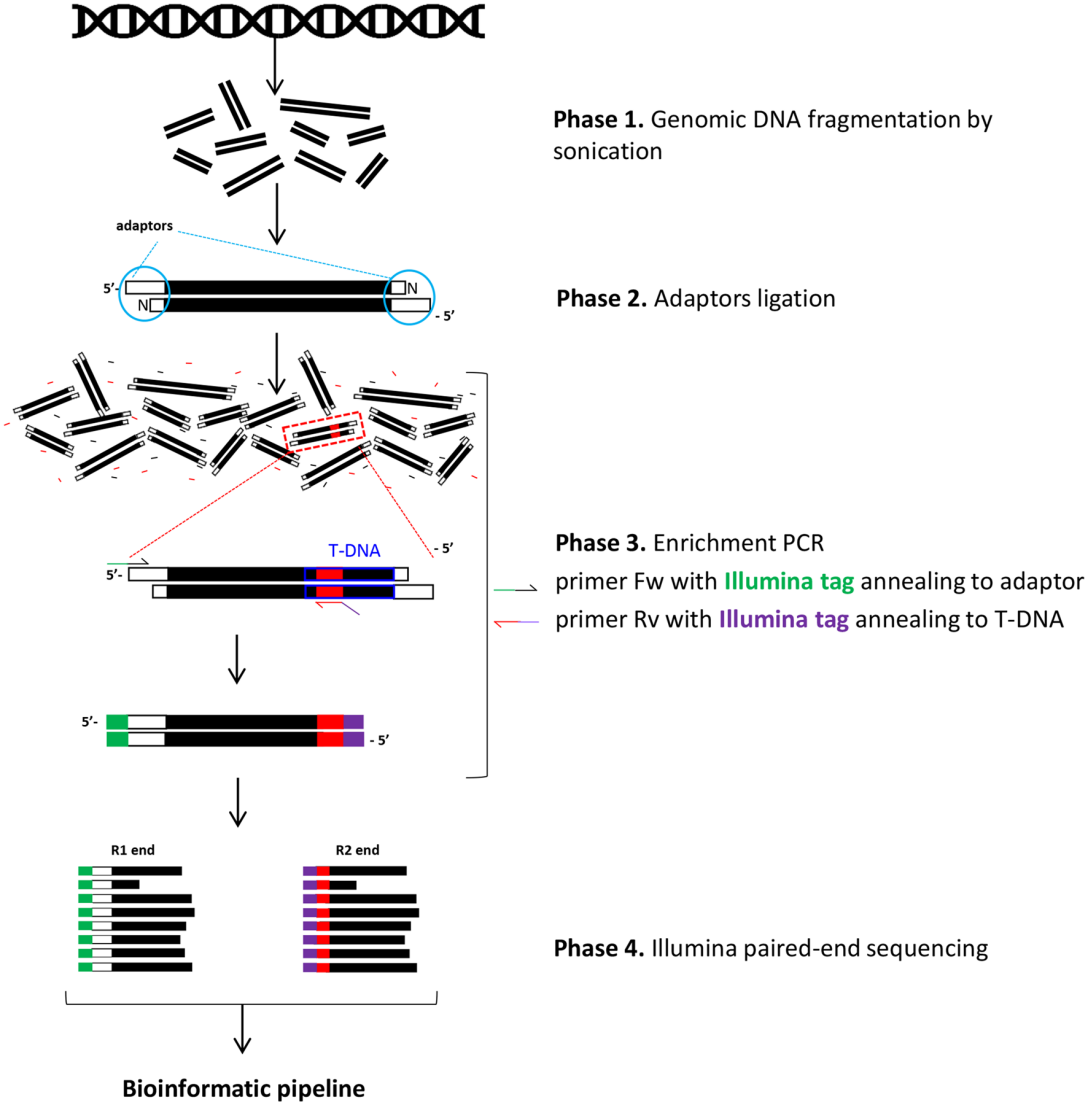
Flow chart of NGS method for the identification of T-DNA integration points in the plant genome. The method is based on sonication of genomic DNA (phase 1) to generate a pool of short DNA fragments which are then ligated to Genome Walker 5′-adaptors (phase 2). A subsequent PCR (phase 3) is performed to amplify DNA fragments containing the junction between the genomic DNA and T-DNA left-end using specific Illumina tagged primers respectively annealing to the 5′-adaptor and to 35S-P. The produced library is sequenced by Illumina paired end sequencing (phase 4) and the obtained raw reads are analyzed by a bioinformatic pipeline as described in the Materials and Methods Section.

The method set up to identify T-DNA position in the plant genome consisted of 4 phases (Fig. 3): (i) fragmentation of genomic DNA; (ii) ligation of adapters to blunt ends of the genomic DNA fragments; (iii) PCR-amplification with primer forward (fw) and reverse (rv) annealing to the 5′-adaptor and to a sequence of T-DNA close to LB respectively; (iv) Illumina paired-end sequencing of the amplified fragments.

In the first instance, we investigated the possibility of detecting the 22 bp-LB sequence left in the plant genome by *Agrobacterium* during the process of T-DNA transfer. Preliminary results on line GT90.1 were unsuccessful. The reads obtained by Illumina sequencing were highly heterogeneous and matched many grapevine genomic regions. Further analysis revealed that this outcome of false positives was due to the presence of a short sequence very similar to the 3′ end of the LB sequence (ATATATCCTG) spanning across the genome and causing unspecific annealing of the LB primer (Table 2).

**Table 2:** Kappamers detected in the genome of Pinot Noir.

Chr. ID	1	2	3	4	5	6	7	8	9	10
n. of hits	587	449	400	517	552	522	501	536	449	415

Chr. ID	11	12	13	14	15	16	17	18	19	
n. of hits	463	516	548	696	449	472	420	721	534	

Data were obtained by blasting the sequence 5′-ATATATCCTG-3′ to the grapevine reference genome from Genoscope (https://www.genoscope.cns.fr/externe/GenomeBrowser/Vitis/)

Chr., Chromosome; n., number

Considering the high risk of amplifying unspecific regions in the third phase of the method, we decided to design a primer rv downstream to the LB site (the distance from LB is 192 bp in vectors 1, 2, 6; 180 bp in vectors 3, 4 and 482 in vector 5) and annealing to the promoter CaMV-P35S (of viral origin), common to all the vectors used in this study. This new strategy was effective, and following the bioinformatic pipeline described in the Materials and Method section, we could identify T-DNA integration point in 11/14 lines of grapevine (78%) and 9/9 lines of apple (100%). We analyzed around 100,000 reads/sample and in some cases, only a few reads could identify the correct T-DNA insertion points. Most of the reads produced were indeed discarded during the merging phase between the two sets of fastq (R1 and R2). The identified genomic regions were then confirmed by an end-point PCR, using a primer fw designed on the genomic DNA and a primer rv on the promoter CaMV-P35S (Supplementary Table 1), followed by Sanger sequencing of the PCR product. For some lines, more than one representative cluster, with a population higher than 10 reads, were found and only the end-point PCR assay allowed discrimination of the true integration region (Table 3). Interestingly, in some cases, the region of integration did not correspond to the most populated cluster.

**Table 3:** Performance assessment of the NGS method for the detection of T-DNA insertion points (PoI) in the plant genome.

Lines	Point of integration (PoI)	Total Reads	No. of clusters	Cluster size of true PoI	Cluster sequence length (bp)
	(Chr. position)				
GT92.2	Chr.14 pos 27434537	136,738	2	69	497
GT103.1	Chr. unknown pos 10269745	97,064	1	71	484
GT109.3	Chr.6 pos 14343411	87,945	1	124	502
GT110.4	Chr.4 pos 20705483	114,260	2	24	481
GT110.5	Not found	57,334	1	53	487
GT110.11	Chr.18 pos 1299053	83,312	1	30	476
GT110.15	Chr.8 pos 17161782	116,919	2	23	450
GT110.18	Chr.19 pos 2014635	141,650	1	176	499
GT110.20	Chr.11 pos 6359640	134,784	1	17	403
V1-4	Chr.05 pos 47471717	201,962	2	30	497
V1-10	Chr.09 pos 36515162	180,599	1	133	487
V1-14	Chr.09 pos 10696063	62,637	1	76	502
V2-3	Chr.00 pos 17491423	91,574	3	758	502
V4-5	Chr.11 pos 5475365	136,756	1	13	486
V4-27	Chr.13 pos 3727444	174,867	3	464	501
V4-34	Chr.01 pos 28464453	123,321	2	25	490
V6-2	Chr.11 pos 9769022	88,950	3	10	475
V6-10	Chr. 5 pos 45547583	72,779	2	32	453

Chr., Chromosome; n., number.

### Features of T-DNA integration in grapevine and apple

**Figure 4:**
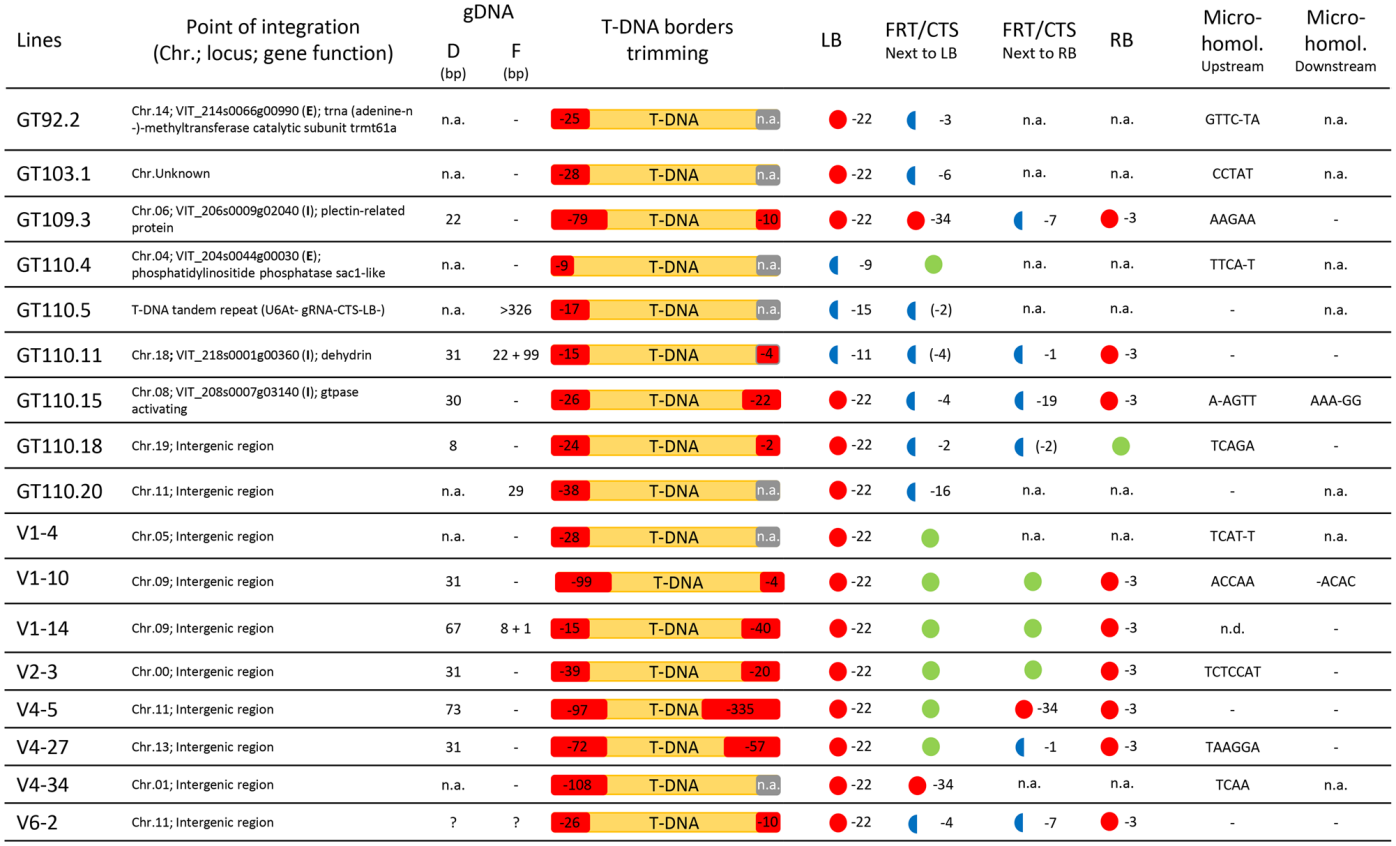
Summary of T-DNA molecular features in grapevine and apple transgenic lines. For each plant line, T-DNA insertion point was identified using the NGS method illustrated in Fig. 3. Trimming at T-DNA borders is reported with red (deletion) or grey (not analyzed, n.a.) boxes. Totally lost, partial or intact elements (LB, FRT/CTS, RB) are respectively indicated with red circle, blue semicircle and green circle. Question mark indicates ambiguities. Exon (E); Intron (I); Chromosome (Chr.); genomic DNA (gDNA); Deletion (D); Filler DNA (F); Micro-homology (Micro-homol.); not detected (n.d.).

(i) *Integration points in genomic DNA* The analysis of the integration point and of T-DNA processing revealed that in two cases, namely for lines GT103.1 and GT103.2 and lines GT110.4 and GT110.8, the lines derived from the same integration event and therefore in Fig. 4 only GT103.1 and GT110.4 lines were reported. Although the sample size of our experiment does not allow us to draw any significant conclusions, we observed that T-DNA integration occurred on different chromosomes in all the analyzed grapevine lines, while two apple lines shared an integration in chromosome 5, two in chromosome 9 and two in chromosome 11, still in different chromosomal regions. Moreover, T-DNA integration points fell in a coding region in five grapevine lines while fell in intergenic regions in apple lines (Fig. 4).
(ii) *Trimming at borders* We determined the number of nucleotides that were lost from the complete LB border (22 nucleotides long) during the integration process. All the grapevine and apple lines showed a trimming of variable size at the LB-end of T-DNA, ranging from − 4 to − 79 bp with an average of − 29 bp in grapevine, and from − 15 to − 108 bp with an average of − 60 bp in apple (Fig. 4). Moreover, for some lines (3 grapevine and 6 apple lines) the RB border was also depicted. Similar to the LB situation, all the lines underwent a trimming at the RB-end too. In grapevine, the deletion ranged from – 2 to − 20 bp with an average of − 11 bp, while for apple the deletion ranged from − 4 to − 335 bp with an average of − 78 bp. This substantial trimming of nucleotides at the borders impacted the LB and RB sequences, as well as the FRT or CTS sequences (with the exception of lines transformed with vector 5), causing a partial or a complete loss of these elements, thus preventing the excision step.
(iii) *Leaky activity* Lines GT110.5, GT110.11 and GT110.18 show small deletions inside the CTS, respectively 2 and 4 nucleotides in the CTS close to LB for lines GT110.5, GT110.11 and 2 nucleotides in the CTS close to RB for line GT110.18, specifically 3 nucleotides apart from the PAM site, the cleavage site of the Cas9 enzyme. Since the analyzed plant replicates were not induced by heat-shock, it may be assumed that the CTS sequence mutation was due to a minimal Cas9 leaky activity even in the absence of heat treatment.
  To better investigate the occurrence of a leaky activity of the inducible heat-shock promoter which control *Cas9* expression, we analyzed transcripts of *Cas9* in biological replicates of grapevine line GT-110-5 induced for 6 h at 42 °C or not induced. In addition, Cas9 and sgRNA expression was checked also in *Agrobacterium* carrying vector 4. Surprisingly, in Agrobacterium cultivated at 28 °C, *Cas9* and sgRNA transcripts were detected (Supplementary Fig. S1A), proving that the plant inducible promoter was recognized by bacterial transcription machinery, but its temperature-control was not functional in this system. Moreover, our results showed that in non-induced plants there is a leaky expression of Cas9 (Supplementary Fig. S1B). To further examine the activity of a hypothetical Cas9-sgRNA complex in Agrobacterium, we checked for T-DNA excision in a bacterial liquid culture. The retention of T-DNA in the binary vector was confirmed because no PCR amplification was detected using primers which bind respectively upstream and downstream to LB and RB (expected size 1,3 Kb) (Supplementary Fig. S1C). In addition, we sequenced the CTSs in 10 independent Agrobacterium colonies and no mutations were detected (Supplementary Fig. S1D). According to these results, we may conclude that a *Cas9*-sgRNA complex can be potentially formed in Agrobacterium, but it is non-functioning since we did not observe either T-DNA excision or CTS mutation. On the contrary, in *planta*, at room temperature, a leaky activity of the heat-shock inducible promoter may be responsible for the mutations found in the CTSs of lines GT110.5, GT110.11, GT110.18.
(iv) *Filler DNA* In some lines we detected the presence of filler DNA between an end of T-DNA and the plant genomic sequence at the insertion site, which is a DNA whose origin is unpredictable (Fig. 4). Lines GT110.11, GT110.20 and V1-14 showed a filler DNA sequence of 22, 29, and 8 bp at the junction between genomic DNA and LB-border while, at the other junction, line GT110.11 showed 99 additional bases. A case of inverted repeats may be ascribed to line GT110.5, where upstream to a partial LB site we found a sequence containing U6At, gRNA, CTS and RB (longer than 326 bp).
(v) *Microhomology* In the lines free of filler unknown DNA, we analyzed the extent of microhomology between T-DNA processed ends (at both LB and RB sides) and the genomic sequence by comparing the pre-insertion genomic DNA sequence from the point of integration in the forward direction, with the exogenous sequence at the border. All the lines, with the exception of apple V4-5 and V6-2, showed upstream (LB-side) a microhomology sequence of 7 bp (V2-3), 6 bp (V4-27), 5 bp (GT-103.1, GT109.3, GT110.18, V1-10) and 4 bp (GT110.3, GT110.15, V1-4, V4-34, V6-10). Downstream (RB-side), less cases were assessed since the sequencing of RB-side (RB processed end and genomic sequence) was only available for a few lines. Microhomology was found in lines GT110.15 (5 bp with a gap in the middle) and V1-10 (4 bp). In many cases there were microhomologous sequences with a gap inside (Fig. 4).
(vi) *Deletion of genomic DNA* Genomic DNA deletions at T-DNA integration sites could be identified only when both sides of T-DNA were sequenced (lines GT109.3, GT110.15, GT110.18, V1-10, V1-14, V2-3, V4-5, V4-27, V6-2). In all of these cases, a deletion of genomic sequences was detected ranging from 8 to 73 bp (Fig. 4) with mean and median values of 36 bp and 31 bp, respectively.

## Discussion

Efficient elimination of the editing machinery after the occurrence of mutation in the target site is a goal of utmost interest because it will likely greatly impact the spread of this technology for crop improvement. In fact, the presence of CRISPR/Cas9 elements in the plant genome strongly increases the risk of off-target effects and, not least, the absence of transgenes is mandatory for complying with legal clues on CRISPRed organisms according to several legislations worldwide. At present, manifold systems have been conceived and tested to isolate transgene-free CRISPRed plants. Despite the delivery of ribonucleoprotein (RNP) complexes via protoplasts transfection produced non-transgenic mutants in *Arabidopsis thaliana*, tobacco and rice^17^, lettuce^18^ and potato^19^, these approaches are limited only to some species due to bottlenecks in the regeneration process for many other crops^20^. Moreover, regeneration of a plant from protoplasts may be risky due to substantial genome instability of *in-vitro* cultured protoplasts. Recently, Fossi et al.^21^, demonstrated that potato plants regenerated from protoplasts were affected by aneuploidy and structural chromosomal changes.

*Agrobacterium*-mediated transformation is actually the most widely used approach to deliver CRISPR/Cas9 components into dicotyledonous plant cells. In the case of sexually propagated plants, T-DNA can be eliminated by Mendelian segregation, resulting in edited but transgene-free progeny^20^. Working with vegetatively propagated and/or highly heterozygous plants, a method to produce non-transgenic edited plants is based on transient CRISPR/Cas9 gene expression. Transient expression of the editing machinery led to an efficiency of 8.2% non-transgenic CRISPRed mutants, when applied on tetraploid tobacco as a model species using the *phytoene desaturase* (*PDS*) gene as a target^22^. A similar approach has been used by Charrier et al.^8^ in apple. Such studies, however, benefit from the albino phenotype induced by the knock-out of the PDS activity, but, without a clear visual marker, the isolation of editing events remains challenging.

Conversely, if *Agrobacterium* stable gene transfer is used, the exogenous DNA removal is advisable for eliminating exogenous sequences and, at the same time, restoring the plant genomic sequence altered by T-DNA integration, especially when this occurred in coding regions.

In this study we assessed and compared the feasibility of two systems for a time-controlled removal of T-DNA following editing in the target site. The first is based on the site-specific recombinase *Flp* that recognizes FRT sites flanking T-DNA borders. This strategy has been applied in many crops to remove the selectable marker gene in view of a cisgenic approach^13,15,23^. In Pompili et al.^12^, this mechanism proved to be successful for the removal of the entire T-DNA cassette in apple. However, in that study, the FRT sites were separated from the LB and RB borders by spacers of 290 and 53 bp respectively (Fig. 1, vector 5). To minimize the amount of exogenous DNA, in this study we tested vectors which did not contain any additional nucleotides between these elements, as thoroughly discussed below. The second system relies on Cas9/CTS: it is completely novel and proposed here for the first time, to our knowledge. This method aims to achieve a simultaneous cleavage of the two CTS with the consequent loss of a long inner fragment (8 kb) before the DNA repair mechanism of the cell is activated. Systems based on dual-sgRNAs targeting two sites on the same chromosome have yet been exploited to mediate deletions of large portions of genomic DNA in some crops. In Arabidopsis, Wu et al.^24^ proved that there is no linear relation between deletions efficiency and deletion size while Ordon et al.^25^, in both *N. benthamiana* and Arabidopsis, observed mainly the occurrence of small deletions (< 100 bp) to the detriment of large deletions. In rice, Wang et al.^26^ achieved deletions of 10 Kb with a frequency of 9% and in soybean Cai et al.^27^ obtained 4.5 Kb-deletions with a frequency of 12%.

Compared to these previous studies, our system has the advantage to rely on two identical synthetic target sites (CTS) and therefore it needs a single sgRNA to produce the deletion, thus overriding the bias due to the putative difference in targeting efficiency of two sgRNAs.

A further key point in the design of the constructs (except vector 5, used as a term of comparison) was the direct connection, without any filling additional DNA, between the LB/RB borders and FRT or CTS elements. This was done in order to leave a minimal trace of exogenous DNA in the plant, once the excision has been accomplished. The presence and length of exogenous DNA in genome edited products are in fact crucial aspects for the definition of their regulatory status. Theoretically, in the first approach, after the correct excision of T-DNA, 1 FRT site of 34-bp should remain in the plant genome, while in the second approach, only 12 bp should remain (in both cases a LB tag of 22 bp and an RB tag of 3 bp have to be considered also) (Fig. 2). On the contrary, the most used commercial binary vectors for plant transformation (Gateway vectors series, pBin series; pCambia series, etc.) present the nopaline LB and RB sequences (24 bp-long) at variable distances from the first downstream or upstream regulatory element (promoter, terminator) in T-DNA. To provide some examples, according to the vector maps publicly available on snapgene website (www.snapgene.com), the size of these spacer DNA close to LB and RB are respectively 85 and 234 nt in Earlygate100 (a gateway vector), 384 and 123 in pBIN19 and 103 and 21 in pCAMBIA0105.

However, many studies in different species demonstrated that T-DNA is not simply inserted into plant genomes as an intact unit. It has been reported that T-DNA can be truncated at the left and/or right ends before the integration^28^. As shown by our results, the processing of T-DNA borders at both ends is common and affected 100% of the lines. This deletion pattern is greater than that observed by Gambino et al.^29^, who analyzed T-DNA-ends in grapevine transgenic lines by inverted PCR and Sanger sequencing. These authors found deletions from 1 to 35 nt at the right border in 14 events out of 22 (63.6%). Regarding the LB, they detected deletions in 17 out of 22 lines (77.3%), ranging from 4 to 60 nt. Likewise, in the framework of a huge analysis of thousands of T-DNA insertion sites in the Arabidopsis GABI-Kat collection, Kleinboelting and colleagues^30^ found that 72.3% and 68% of the lines were deleted respectively at LB and RB borders. Truncated T-DNA border regions were also frequently found in apple^31,32^. In order to remove selectable marker gene via site-specific recombination, Timerbaev and colleagues^33^ analyzed three independent apple lines and observed the loss of the recombination site close to the left border in two of them. Similarly, our results showed that the frequency and the extent of the trimming process at T-DNA borders was deleterious for the maintenance of those elements crucial for T-DNA cassette removal. Only the lines transformed with vector 5 (the vector with filler DNA flanking borders) preserved both FRT elements intact, while for the lines transformed with the other vectors only GT110.4 showed one entire CTS out of two. In fact, in this line (GT110.8 is the same event) we were unable to identify and sequence the RB-end, probably because of genomic rearrangement (two PCR assays, aimed at amplifying different length of the junction region between T-DNA RB-end and genomic DNA, were negative, data not shown). Moreover, this such hypothesis is supported by the fact that no editing was observed in the target sites in those lines (Table 1), due, presumably, to a rearrangement involving the sequence of Cas9 itself or of sgRNA which resulted in the non-functionality of the editing machinery.

Our results, both in grapevine and in apple, showed a predominance of microhomology sequences at junctions between T-DNA and the host genomic DNA, as well as DNA deletions of genomic DNA and presence of filler DNA. This is in agreement with previous studies conducted in grapevine^29^ and apple^32^. Such T-DNA integration signatures are consistent with the polymerase-θ-mediated mechanism of DSB repair as described by Van Kregten et al.^34^ and Gelvin^35^. These authors demonstrated that polymerase-θ captures T-DNA’s 3′ end (LB site) at genomic DSB through microhomology sequences between T-DNA and plant DNA.

We also observed leakage activity of Cas9 prior to heat-shock induction in lines GT110.5, GT110.11 and GT110.18 which showed partially deleted and therefore non-functional CTS. A basal expression of Cas9 (not yet induced by the treatment) was likely responsible for the DNA cleavage at CTS (between the third and the fourth nucleotide from the PAM site) with the outcome of some small deletions. Nandy and colleagues^36^, who applied heat‐shock-inducible CRISPR/Cas9 system in rice, detected a rate of basal targeted mutagenesis (before HS) around 16% (two out of 12 lines showed editing in one target site; 1 out of 6 lines in a second target site). This was quite unexpected because when used to drive expression of recombinase enzymes, the soybean heat-shock promoter proved to be tightly regulated by heat treatment^15,23^.

Another interesting result of this study is the low targeting efficiency of HS-inducible Cas9 compared to constitutive Cas9 (Table 1). This finding was also appreciated by Nandy et al.^36^, who compared the relative expressions of HS inducible- versus constitutive-Cas9 and found that the latter was 800 times more expressed than the former. The low expression level of inducible Cas9 can be partially balanced by its higher enzymatic activity at the high temperature used during the induction step, close to 40°C^37^.

A large part of our work regarded the setup of a NGS method to characterize the integrity of T-DNA border sequences, crucial for the excision of T-DNA cassette. Next-generation sequencing (NGS) technologies offer rapid and cost-effective options for detecting the genomic location of T-DNA, as well as for identifying nucleotides variation (SNP, deletions, insertion) in the junctions between genomic DNA and T-DNA. Different NGS applications have been deployed over the past years for transgenic lines characterization. Some groups have carried out a demanding whole genome sequencing, able to depict all possible kinds of variations caused by the process of transformation even at long distances from T-DNA integration site. There are examples in the model plant *Arabidopsis* as well as in important crops like rice, soybean, maize^38–41^. Other studies made use of a target enrichment step to focus the NGS analysis on the specific region of interest. Among these are applications based on the use of biotinylated primers, complementary to T-DNA, combined with streptavidin beads to capture hybridized sequences^38,42,43^. Alternative methods relied on the ligation of fragmented DNA with adapters followed by a PCR with one primer annealing to T-DNA and the other primer annealing to adapter^44–46^. The method we set up belongs to this category, but contrasts with the strategy employed by the SALK Institute to define insertion sites in an Arabidopsis mutants collection^44,46^, based on the digestion of genomic DNA with a restriction enzyme. In fact, we fragmented the DNA by sonication in order to have DNA fragments of similar size (ranging from 200 to 1000 bp) and unaffected by the nucleotide sequence. At first, we focused on seeking the LB sequence (by using a reverse primer annealing to LB in phase 3 of the method) that in theory would be present in all the organisms modified via *Agrobacterium*, like a specific footprint proving the biotechnological origin of the product. However, using this approach, we were not successful due to borders trimming with the consequent loss of LB element. This became clear when we identified and sequenced T-DNA integration points in our plants by using a more internal primer. The optimized method identified the location of T-DNA in the genome of 78% of grapevine lines and 100% of apple lines, though it can be further improved by a higher fragmentation of the genomic DNA to reduce the number of discarded reads during the merging phase.

## Conclusions

Our study showed that the trimming frequency of T-DNA borders, which leads to total or partial degradation of FRT and CTS sequences, is very high. It also showed that the heat-shock inducible promoter we used is prone to a basal leaky activity of the Cas9 enzyme, sufficient to mutate and inactivate a CTS sequence. While Pompili et al.^12^ demonstrated that if FRT sites are separated from LB and RB by spacer DNA, the removal of T-DNA cassette can be achieved efficiently, to evaluate the applicability of the second system based on Cas9/CTS without the presence of any spacer DNA, a large number of plants should be assessed. Our results proved that the design of vectors aimed at obtaining transgene-free CRISPRed fruit trees needs to take into consideration many factors associated with the mechanism of T-DNA integration in the plant cell. Considering the long time required to transform grapevine and apple and the low efficiency of the processes, the identification of transgene-free edited plants will require a large effort in terms of the number of regenerated plants and genotyping analysis.

## Methods

### Binary vectors design

The binary vectors used to transform grapevine and apple cultivars were conceived by us (Fig. 1 and Fig. 2) and assembled by DNA Cloning Service (Hamburg, Germany). The nucleotide sequence of *SpCas9* and of *nptII* gene were codon optimized for the plant expression system and their sequences are available on the company website (https://www.dna-cloning.com/). The heat-shock inducible promoter is from the soybean gene *Hsp17.5-E*^47^. The sequence of FRT site was 5′-gaagttcctatactttctagagaataggaacttc-3′. In vector 3 the sequence of the CTS next to LB was 5′-**cct**cagagcatgtgttcttaaaa-3′ (in bold the PAM site) and that of the CTS next to RB was its reverse complement (5′- ttttaagaacacatgctctg**agg**-3′). In vector 4 the sequence of the CTS next to LB was 5′-**cct**cagagttgcctcctttcccc-3′ and that of the CTS next to RB was its reverse complement (5′-ggggaaaggaggcaactctg**agg**-3′). The sequences of the guide RNAs carried by the vectors were: (vector 1) 5′-CACTTGGCACCCTTGTAAAA-3′ for the targets 1 in exon no. 3 of *VvMLO7*; (vector 2) 5′-CACTTGGCACCCTTGTAAAA-3′ (gRNA 1) and 5′-TTTTAAGAACACATGCTCTG-3′ (gRNA 2) for the target 1 and 2 in exon no. 3 of *VvMLO7*; (vector 3) 5′-TTTTAAGAACACATGCTCTG-3′ (gRNA 2) for the target 2 in exon no. 3 of *VvMLO7*, repeated twice; (vector 4) 5′-ggggaaaggaggcaactctg-3′ for the target in *VvIdnDH*^16^ and 5′-TTTTAAGAACACATGCTCTG-3′ (gRNA 2) for the target 2 in exon no. 3 of *VvMLO7*; (vector 5) 5′-GCTGTATTCCGCATGAATCC-3′ for the target of exon no. 2 of *MdDIPM4*; (vector 6) 5′-GCTGTATTCCGCATGAATCC-3′ (gRNA 1) for the target of exon no. 2 of *MdDIPM4* and 5′-CGATTGGCTGGTGAGGTAAT-3′ (gRNA 2) for the target of exon no. 1 of *MdDIPM1*.

### Gene transfer experiments

For grapevine, *Agrobacterium tumefaciens* (*A.t.*)-mediated gene transfer was performed on embryogenic calli of Chardonnay, Thompson seedless, Microvine 04C023V0006 (derived from a cross between “Grenache” and the original L1 mutant microvine^48^), and Sugraone genotypes according to Dalla Costa et al.^49^. Several experiments were carried out using *A.t.* strain EHA105^9^ carrying vectors from 1 to 4 described in Fig. 1. *NptII* was used as selectable marker to confer resistance to kanamycin. For apple, *A.t.* strain EHA105 carrying vectors from 5 to 6 (described in Fig. 1) was used to transform plantlets of *Malus x domestica*, cultivars ‘Gala’ and ‘Golden Delicious’, as described by Joshi et al.^50^. Grapevine lines were obtained after 9–12 months from co-culture with *A.t.*, while apple lines after 6–7 months. Regenerated plants were screened for the presence of *SpCas9* (to select plants which integrated T-DNA) and *VirG* (to exclude the persistence of *A.t.* in the plant) by PCR in 20 µl final volume containing 1 × PCR BIO (Resnova, Rome, Italy), 0.5 µM of each primer (SpCas9_Fw: 5′-AGATCCTCACTTTTAGAATCCC-3′ and SpCas9_Rv: 5′-TGTCCTTGATAATCTTCAGGAG-3′, amplification products: 486 bp; VirG_Fw: 5′-GCCGGGGCGAGACCATAGG-3′ and VirG_Rv: 5′-CGCACGCGCAAGGCAACC-3′, amplification products: 605 bp) and 40 ng of genomic DNA. DNA was extracted from freshly frozen leaf tissue (approximately 100 mg) using Nucleospin Plant II kit (Macherey–Nagel, Düren, Germany) following the manufacturer’s instruction, quantified using Nanodrop 8800 (Thermo Scientific) and diluted to a final concentration of 20 ng/µL.

### Transgene copy number quantification

The real-time PCR quantification of *nptII* or *SpCas9* copy number (CN) in grapevine and apple lines was carried out according to real-time PCR methods developed by Dalla Costa et al. for grapevine^51^ and apple^52^. Reactions were performed in a 96-well plate on a C1000 thermal cycler (Bio-Rad, Hercules, USA) equipped with CFX96 real-time PCR detection system (Bio-Rad, Hercules, USA). The real-time PCR singleplex reaction was carried out in a 10 µl final volume containing 1 × SsoAdvanced Universal Probes Supermix (Bio-Rad, Hercules, USA), 40 ng of genomic DNA, 0.3 µM primers (Sigma, Haverhill, UK) and a 0.2 µM specific Taqman probe (Sigma, Haverhill, UK). The thermal protocol was as follows: polymerase activation for 3 min at 95 °C followed by 40 cycles of denaturation of 10 s at 95 °C, annealing of 5 s at 58 °C and 5 s at 60 °C and an elongation of 30 s at 72 °C. Primers and Taqman probes used to amplify grapevine endogenous gene *chi*^51^ and apple endogenous gene *Topo6*^52^ were: *VvChi*_fw:5′-GAGGCTGGGGATGAGAAAATTG-3′, *VvChi*_rv:5′-CCCATCTCTCCTTCAACCACCT-3′, ′*VvChi*_Probe: FAM-5′-AAGCTGAGAAGGTTGCTCCGGT-3′-TAMRA (amplification product size: 75 bp); *MdTopo6*_fw: 5′-TGTGGAAGGAGATCAAAGCGCA3′, *MdTopo6*_rv:5′-CGCGTTGCTTCTTTGCTGCA-3′, *MdTopo6*_Probe: FAM-5′-ACATGCCAACAGGAACAATCACA-3′-TAMRA (amplification product size: 196 bp). Primers and TaqMan probes for *nptII* and *SpCas9* were: *nptII*_fw: 5′-CTTGCCGAATATCATGGTGGAA-3′, *nptII*_rv: 5′-GGTAGCCAACGCTATGTCCTGA-3′, *nptII*_Probe: FAM-5′-TTCTGGATTCATCGACTGTGGC-3′-TAMRA (amplification product size: 101 bp); *SpCas9*_fw: 5′- TACGCTGACCTTTTCTTGG-3′, *SpCas9*_rv: 5′-CTTGGTGATCTCAGTGTTCA-3′, *SpCas9*_Probe: FAM-5′- CCTCTCCGACGCTATTCTGCTCTCC-3′ (amplification product size: 87 bp). The standard curves (four points, starting from 10^6^ plasmid molecules and adopting a serial dilution of 1:5) were built with a plasmid pGEM-T easy (Promega, Madison, Wisconsin, USA), named universal plasmid calibrator, in which we cloned a fragment of each of the four genes quantified (*VvChi*, *MdTopo6*, *nptII* and *Cas9*). For each sample, the transgene (*nptII* or *SpCas9*) CN was calculated using the following formula: (transgene total copies/endogenous gene total copies) × 2. The total copies of transgene and endogenous gene were calculated on the basis of the mean values of the quantification cycles (Cq) of two technical replicates.

### On-target editing evaluation

A region of the gene *VvMLO7* (between exon no. 2 and intron no. 3, containing the target sites of the CRISPR/Cas9 system) was amplified with primer fw 5′-GCGAGCCCTTTATGAATCTCTGG-3′ and primer rv 5′-CTGCAGGAAAAGAGGGTGGGAA-3′ (amplification products: 367 bp). *MdDIPM1* and *MdDIPM4* regions containing the target sites were amplified with primers *MdDIPM1*_fw: 5′-CTGTATAGCTGAGCCTCCCC-3′ and *MdDIPM1*_rv: 5′-CCACAAGAAAACTGGCGTCA-3′ (amplification product: 324 bp) and primers *MdDIPM4*_fw: 5′-GTGTTCAGTTTGGGGCACAT-3′ and *MdDIPM4*_rv 5′-GGAGGTTCTAACGGGGAGAG-3′ (amplification product: 399 bp). PCR was carried out in 20 µl final volume containing 1 × PCR BIO (Resnova, Rome, Italy), 0.4 µM of each primer (both elongated with overhang Illumina adapters) and 50 ng of genomic DNA. The Illumina library was sequenced on an Illumina MiSeq (PE300) platform at the Sequencing Platform Facility of Fondazione Edmund Mach (San Michele all’Adige, Italy). The CRISPResso pipeline (https://crispresso.rocks/) was used to process (with default parameters) the raw paired-end reads and to visualize the mutations profiles in the target sequences.

### Heat-shock induction strategies

Three strategies of heat-shock induction were carried out in grapevine lines. The first (A) consisted in three incubations of baby jars containing 3-week old plantlets at 42 °C for 6-h with a 42-h interval between consecutive heat treatments. The second (B) and the third (C) strategies consisted in incubations (three for B and five for C) of petri dishes containing buds at 42 °C for 3-h with a 21-h interval between consecutive heat treatments. Petri dishes were laid between preheated petri dishes containing water. During heat-shock assays, plantlets (two biological replicates for each line) and buds (tree for each line and induction strategy) were maintained in WP medium^53^. Three nodes from induced plantlets as well as induced buds were micro-propagated in WP medium in baby jars and, after 1 month, 2 central leaves were collected from the regenerated plantlets for DNA extraction and T-DNA quantification. For apple, 2-week old plants were incubated three times at 42 °C for 6-h with a 48-h interval between consecutive incubations. At the end of the heat-shock inductions, leaves, the vegetative apex and the first 1–2 basal internodes of each plant were discarded. The 2 central nodes of the stem were collected and placed horizontally onto a fresh propagation medium to promote the regeneration of new shoots. After 1 month, the first 2 leaves of 10 regenerated shoots for line were collected for DNA extraction and *nptII* quantification. Heat incubations of baby jars or petri dishes containing plantlets or buds were carried out in preheated hybridization oven hybridizer HB-1000 (UVP, Upland, CA). After the incubations, jars or petri dishes were returned to the tissue culture chamber set at 25 °C for further growth.

### NGS method for T-DNA integration site identification and bioinformatics pipeline

The method consists in 4 phases as illustrated in Fig. 3. *Phase 1. Genomic DNA fragmentation*: 1 µg of DNA for each sample was fragmented using BIORUPTOR NextGen (Diagenode, Seraing, Belgium) with 3 cycles of 30 s at low intensity to obtain fragments ranging between 200 and 1000 bp. DNA fragmentation profile was checked on Tapestation 2200 (Agilent, Santa Clara, CA, USA) using D1000 or Genomic Dna ScreenTape. Fragmented DNA was purified with Ampure XP beads (Beckman, Brea, CA, USA ) at 1.8 × ratio, treated with NEBNext End Repair Module E6050S (New England Biolabs, Ipswich, MA, USA) and again purified with 1.8 × Ampure XP beads. Final amount of purified end-repaired fragments was checked with D1000 ScreenTape on Tapestation. *Phase 2. Adaptors ligation*: genomic blunt fragments were ligated to the Adaptors of the Universal GenomeWalker 2.0 kit (Takara Bio, Kusatsu, Japan) using T4 Ligase (Thermo Scientific, Waltham, MA, USA) and following manufacturer instruction. *Phase 3. Enrichment PCR*: PCR was performed in a 20 µl final volume containing 1 × PCR BIO (Resnova, Rome, Italy), 0.25 µM of the following primers, ADAP_ill: 5′-*GTCTCGTGGGCTCGGAGATGTGTATAAGAGACAG*GTAATACGACTCACTATAGGGC-3′; P35S_ill: 5′-*TCGTCGGCAGCGTCAGATGTGTATAAGAGACAG*GCTGGGCAATGGAATCCGAG-3′ (the sequence in *italics* is the Illumina index adapter) and 20 ng genomic DNA. The PCR product was purified with 0.8 × AMPure XP Beads to remove fragments smaller than 200 bp, primers and primer dimers. *Phase 4. Illumina paired-end sequencing:* the library was sequenced by Illumina MiSeq (PE300) platform at the Sequencing Platform Facility of Fondazione Edmund Mach (San Michele all’Adige, Italy). Approximately 100,000 reads per sample were produced. *Bioinformatic pipeline*: two datasets of raw sequencing reads (fastQ files for both ends) were analyzed using VSEARCH 2.13.4^54^ and Blast 2.9.0 (https://blast.ncbi.nlm.nih.gov/Blast.cgi) software. Reads of Dataset 1 (amplified with the primer ADAP_ill) were trimmed of 48 bp to remove the GenomeWalker adaptor sequence and then merged with the reads of dataset 2 (minimum overlapping = 50 bp); merged sequences were then clustered using an identity threshold (ID) minimum of 0.90. To identify exogenous sequences, clusters were mapped to T-DNA vector sequence using the Blast tool and filtered according to the alignment length (> 50 bp) and e-value above 0.01. Filtered sequences were then mapped against the reference genome, and hits with less than 10 mismatches and an e-value above 10^–6^ were selected. According to blast output, specific genomic regions were identified, corresponding to T-DNA integration points. T-DNA locations were confirmed by PCR amplification of regions covering the upstream and downstream junctions between genomic DNA and T-DNA. PCR was performed in a 20 µl final volume containing 1 × PCR BIO (Resnova, Rome, Italy), 50 ng of genomic DNA and 0.5 µM of the primers reported in Supplementary Table 1. Amplification products were checked on agarose gel, purified using PureLink Quick Gel Extraction (Invitrogen, Carlsbad, CA, USA) or PCR Purification Combo Kit (Thermo Scientific, Waltham, MA, USA) and sequenced by Sanger sequencing (FEM Sequencing Platform Facility). Sequencing output was analyzed with Blast online tool (blast.ncbi.nlm.nih.gov).

### Checking for the leaky activity of the soybean heat-shock promoter

Gene expression analysis: total RNA was isolated from *Agrobacterium tumefaciens* liquid colture (4 ml; OD = 0.6) using the lysozyme-Trizol method as describe in Villa-Rodríguez^55^, and from grapevine leaves using the Spectrum Plant Total RNA Kit (Sigma Aldrich, St. Louis, MO, USA). RNA was quantified with the spectrophotometer NanoDrop ND-8000 (NanoDrop Technologies, Wilmington, DE, USA) and by gel electrophoresis. Following DNase treatment, 1 µg of RNA was retrotranscribed into cDNA with the SuperScript III Reverse Transcriptase (Invitrogen, Carlsbad, CA, USA) and random primers. The Real-time PCR was carried out on the CFX96 instrument (Bio-rad, Hercules, CA, USA) in 12.5 µl volume containing SsoAdvanced Universal SYBR Green Supermix (Bio-rad ,Hercules, CA, USA), 0.5 µM primers (Supplementary Table 2) and 1 µl of diluted cDNA (1:10). An initial denaturation step at 95 °C for 5 min was followed by 40 cycles at 95 °C for 10 s and 60 °C for 30 s. Finally, to detect non-specific amplification in cDNA samples, a melting curve analysis was performed as follows: 95 °C for 10 s, 65 °C for 5 s and a stepwise T increase (0.5 °C/s) up to 95 °C with a continuous detection. Glyceraldehyde 3-phospate dehydrogenase (GAPDH) and spectinomycin (Supplementary Table 2) were used as housekeeping genes, to determine the initial quantity of cDNA in grapevine and Agrobacterium respectively.

Colony PCR: individual colonies of *Agrobacterium tumefaciens* were added directly to the PCR reaction mix by an inoculation loop. PCR amplifications were performed in a final volume of 20 µl containing PCRBIO Taq Mix Red (PCR Biosystems Ltd., London, UK) and 0.5 µM primers (Supplementary Table 2) using the Thermocycler Tgradient (Biometra, Gottingen, Germany). The PCR consisted of an initial denaturing step of 2 min at 95 °C followed by 35 cycles of denaturation, annealing and extension of 15 s at 95 °C, 20 s at 60 °C and 60 s at 72 °C respectively, with a final extension of 5 min at 72 °C. PCR products (5 µl) were separated by electrophoresis at a constant voltage (100 V) on a 1.2% agarose gel (Sigma) stained with Ethidium bromide and visualized by Gel Doc 2000 (Biorad, Hercules, CA, USA).

Sanger sequencing: the products of the colony PCR for the CTS screening of the 10 individual *Agrobacterium* colonies (strain EHA105 carrying vector 4) were purified with magnetic beads using the CleanNGS kit (CleanNA, Waddinxveen, The Netherlands) and sequenced by Sanger sequencing (FEM Sequencing Platform Facility).

## Supplementary information

Supplementary Figure 1.

Supplementary Table 1.

Supplementary Table 2.

## Data Availability

The datasets generated and/or analyzed during the current study are available from the corresponding author on reasonable request.
