# Supplementary material for: Strategies to produce T-DNA free CRISPRed fruit trees via Agrobacterium tumefaciens stable gene transfer
Source: Sci Rep. 2020 Nov 19;10:20155. doi: 10.1038/s41598-020-77110-1 (PMC7678832; doi:10.1038/s41598-020-77110-1)
Supplement: Supplementary file 1 — Supplementary Figure 1. [file 41598_2020_77110_MOESM1_ESM.docx]

**Title**

**Strategies to produce T-DNA free CRISPRed fruit trees via *Agrobacterium tumefaciens* stable gene transfer**

**Authors**

Lorenza Dalla Costa^1,∞,^*, Stefano Piazza^1,∞^, Valerio Pompili^1^, Umberto Salvagnin^1^, Alessandro Cestaro^1^, Loredana Moffa^1^, Lorenzo Vittani^1^, Claudio Moser^1^ & Mickael Malnoy^1^

^
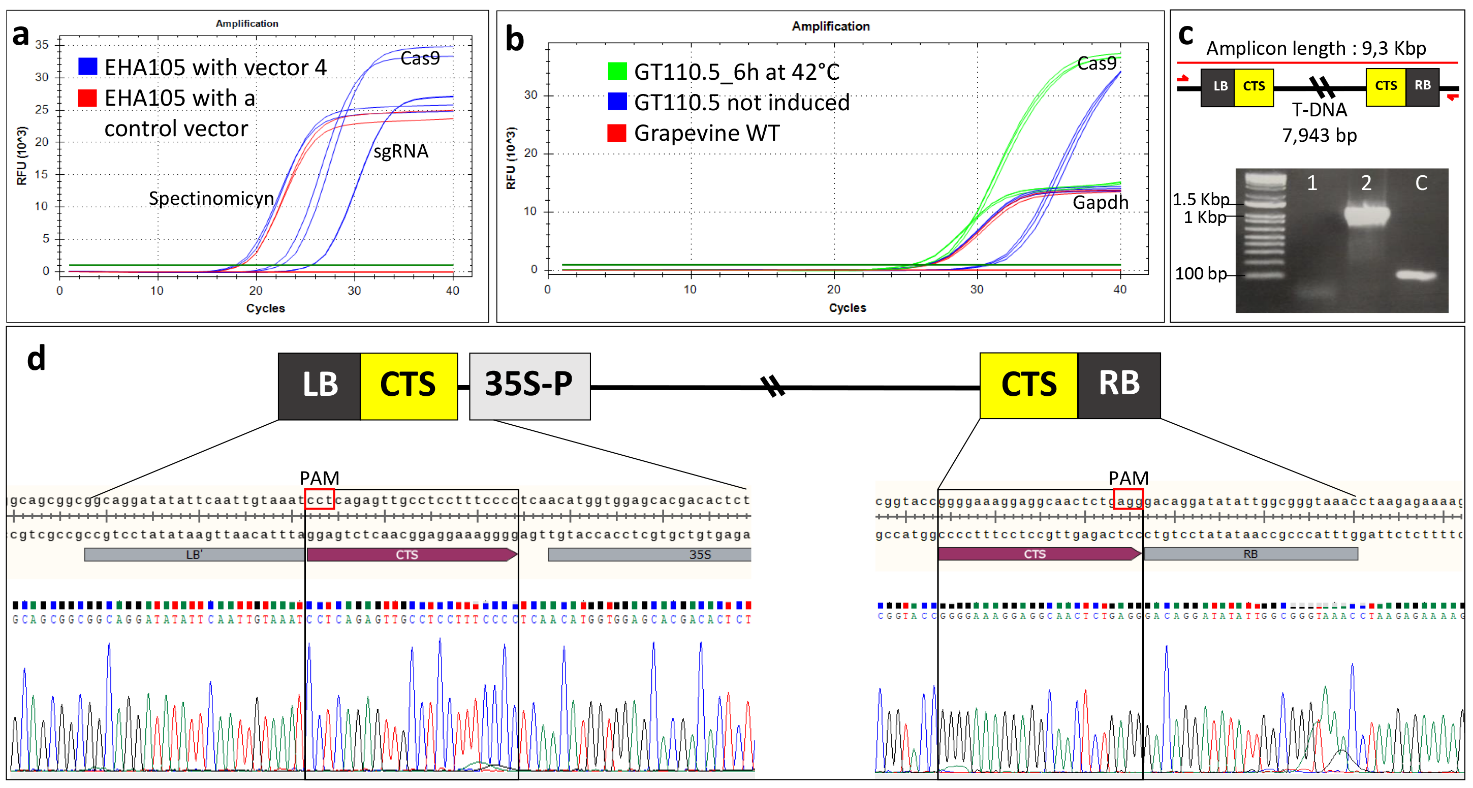
^

**Supplementary Figure 1**. Analysis of the leaky activity of the heat shock promoter. A) Expression analysis of *Cas9* and sgRNA in two stocks of *Agrobacterium tumefaciens* EHA105, one carrying vector 4 (in blue) and the other (in red), used as control, carrying a vector for the overexpression of a reporter gene. B) Expression analysis of *Cas9* in grapevine line GT110.5 induced (in green) or non-induced (in blue) by heat treatment. C) PCR evaluation of the T-DNA cassette excision using primers which anneal outside the T-DNA (drawn in red); lane 1: *Agrobacterium tumefaciens* (*A.t*.) EHA105 carrying vector 4 (1 ul of liquid culture); lane 2 = control vector, where T-DNA has been removed by restriction enzymes digestion (expected size: 1325 bp); lane C: as for control, *A.t.* EHA105 carrying vector 4 (1 ul of liquid culture) was amplified using primers which anneal to spectinomycin resistance gene on the backbone (expected size: 122 bp). D) Sequencing results of the CTS in 10 independent colonies of *A.t.* EHA105 carrying vector 4. All the CTS were identical to the expected reference sequence (one example is shown).
