# Supplementary material for: Strategies to produce T-DNA free CRISPRed fruit trees via Agrobacterium tumefaciens stable gene transfer
Source: Sci Rep. 2020 Nov 19;10:20155. doi: 10.1038/s41598-020-77110-1 (PMC7678832; doi:10.1038/s41598-020-77110-1)
Supplement: Supplementary file 2 — Supplementary Table 1. [file 41598_2020_77110_MOESM2_ESM.docx]

**Title**

**Strategies to produce T-DNA free CRISPRed fruit trees via *Agrobacterium tumefaciens* stable gene transfer**

**Authors**

Lorenza Dalla Costa^1,∞,^*, Stefano Piazza^1,∞^, Valerio Pompili^1^, Umberto Salvagnin^1^, Alessandro Cestaro^1^, Loredana Moffa^1^, Lorenzo Vittani^1^, Claudio Moser^1^ & Mickael Malnoy^1^

| **ID line** | **Upstream junction: fw primers used in combination with a unique rv primer complementary to P35S promoter (5'-GCTGGGCAATGGAATCCGAG-3'** | **Downstream junction: rv primers used in combination with a unique fw primer complementary to NOS terminator (5'-CGCGCGGTGTCATCTATGTT-3')** |
| --- | --- | --- |
| GT-92.2 | 5'-TCCAGAAGCTACCCCAGTTACA-3' |  |
| GT-103.1 (and GT-103.2) | 5'-AGCTCCCTATTTCTCTGCAACT-3' |  |
| GT-109.3 | 5'-CCTCATCCTTTCCTCCACCCCA-3' | 5'-AGTTGCAAAGGAAAAGGTGGGA-3' |
| GT-110.4 (and GT-110.8) | 5'-CTTTCCACCACTCCCCACTGTC-3' |  |
| GT-110.11 | 5'-TCTAGAACCAAACATGACCTTCAA-3' | 5'-GATGCAACCGAAAGCCACTT-3' |
| GT 110.15 | 5'-CGGCCTCCCTGTTGAGTTTGAA-3' | 5'-TGAGAGGGAGAAGGAATATTTCG-3' |
| GT 110.18 | 5'-AGAAAGAACGAGACAGTCACAGCA-3' | 5'-CCTTGAAATGTCCCGTAGCA-3' |
| GT-110.20 | 5'-TCATCCCATTCTTAACAGTCTCGCA-3' |  |
| V1.4 | 5'-TCTCAGGCTATCGGTTTCAAGTCC-3' |  |
| V1.10 | 5'-GATCGCCACAGTTCCAATTCCCA-3' | 5'-TACGCAGCCACCTTTTCAAC-3' |
| V1.14 | 5'-CGTAGTGGTTTGGGGTTGATGAC-3' | 5'-AAATTTGCAAGTGACGCCCA-3' |
| V2.3 | 5'-GACCCCTCATCGATGGTTGGTTC-3' | 5'-GCCAGCACCACCAAGTTTTA-3' |
| V4.5 | 5'-CCGTGTTTAGCTGGTTTCCTCGT-3' | 5'-GTGCCTCAAACGAGCATGTT-3' |
| V4.27 | 5'-AGGTGGATTTTGTGGAAGAGATTCA-3' | 5'-ACCACACGCTCCACTAGTAC-3' |
|  | 5'-TCCTTGCGCGATGACAATCTTCT-3' |  |
| V4.34 | 5'-GCCTTGTTCATCATCCGCACTCT-3' |  |
| V6.2 | 5'-TCTTGGCCTCTCTCGTTTTCTCAC-3' | 5'-GTACAACGTGCATGGTGGAG-3' |
| V6.10 | 5'-GGTTCATTTGCCCACGTTCTTACA-3' |  |

**Supplementary Table S1.** Primers used to validate the T-DNA integration points identified with the NGS method
