# Supplementary material for: Strategies to produce T-DNA free CRISPRed fruit trees via Agrobacterium tumefaciens stable gene transfer
Source: Sci Rep. 2020 Nov 19;10:20155. doi: 10.1038/s41598-020-77110-1 (PMC7678832; doi:10.1038/s41598-020-77110-1)
Supplement: Supplementary file 3 — Supplementary Table 2. [file 41598_2020_77110_MOESM3_ESM.docx]

**Title**

**Strategies to produce T-DNA free CRISPRed fruit trees via *Agrobacterium tumefaciens* stable gene transfer**

**Authors**

Lorenza Dalla Costa^1,∞,^*, Stefano Piazza^1,∞^, Valerio Pompili^1^, Umberto Salvagnin^1^, Alessandro Cestaro^1^, Loredana Moffa^1^, Lorenzo Vittani^1^, Claudio Moser^1^ & Mickael Malnoy^1^

| **Primer for gene expression analysis**  **(Real-time PCR)** | **Sequence 5’ -> 3’** | **Amplicon lenght** |
| --- | --- | --- |
| GAPDH fw | TTCTCGTTGAGGGCTATTCCA | 70 bp |
| GAPDH rv | CCACAGACTTCATCGGTGACA |  |
| Spectinomycin fw | ATCATTCCGTGGCGTTATCC | 122 bp |
| Spectinomycin rv | GTCAGCAAGATAGCCAGATCAA |  |
| Cas9 fw | AAGTCCGAGGAAACCATCACC | 117 bp |
| Cas9 rv | TTTCTCGTTGGGCAGGTTCTT |  |
| idtSgRNA fw | GGGGAAAGGAGGCAACTCTG | 92 bp |
| univSgRNA rv | CGACTCGGTGCCACTTTT |  |
| **Primer for checking T-DNA excision in Agrobacterium (colony PCR)** | **Sequence 5’ -> 3’** | **Amplicon lenght** |
| Spectinomycin fw | ATCATTCCGTGGCGTTATCC | 1325 bp |
| Downstream RB rv | TGGTTGGCATGCACATACAAA |  |
| **Primer for screening of CTS (colony PCR)** | **Sequence 5’ -> 3’** | **Amplicon lenght** |
| Spectinomycin fw (CTS next to LB) | ATCATTCCGTGGCGTTATCC | 1207 bp |
| P35S promoter rv (CTS next to LB) | GCTGGGCAATGGAATCCGAG |  |
| NOS terminator fw (CTS next to RB) | CGCGCGGTGTCATCTATGTT | 1351 bp |
| Downstream RB rv (CTS next to RB) | TGGTTGGCATGCACATACAAA |  |

**Supplementary Table 2**. List of primers used in Real-time PCR and colony-PCR during the examination of the leaky activity of the heat-shock promoter
